# Evaluating the impact of distance learning on gender-affirming healthcare competence: knowledge acquisition and satisfaction among healthcare professionals in Italy

**DOI:** 10.3389/fpubh.2024.1393188

**Published:** 2024-06-06

**Authors:** Ughetta Maria Favazzi, Matteo Marconi, Pietro Carbone, Debora Guerrera, Angela Ruocco, Martina Manoli, Francesca Molinaro, Federica Maria Regini, Andrea Vittozzi, Alfonso Mazzaccara, Marina Pierdominici

**Affiliations:** ^1^Training Office, Istituto Superiore di Sanità, Rome, Italy; ^2^Reference Centre for Gender Medicine, Istituto Superiore di Sanità, Rome, Italy; ^3^Department of Medical and Surgical Sciences and Advanced Technologies “GF Ingrassia”, University of Catania, Catania, Italy

**Keywords:** transgender, healthcare, distance learning, healthcare professionals, training, knowledge, Problem-Based Learning

## Abstract

**Background:**

Transgender and gender diverse (TGD) individuals face significant healthcare barriers, with one of the most critical being the inadequate knowledge and skills of healthcare professionals (HCPs) in TGD health. To address this issue, we undertook a project to develop a distance learning course for all healthcare professions, encompassing a comprehensive range of topics related to TGD health issues.

**Objectives:**

This study aimed to evaluate the impact of a course on gender-affirming healthcare competence, with a focus on knowledge acquisition and satisfaction levels. The hypothesis was that participating in the course would enhance the participants' knowledge on the covered topics.

**Methods:**

A distance learning course, designed for all Continuing Medical Education professions, was conducted between March and September 2023. The course was structured according to the Problem-Based Learning methodology. We implemented a pre-test vs. post-test study design to evaluate the enhancement of knowledge, based on a set of Multiple Choice Questions (MCQs), and investigated users' satisfaction through the administration of a semi-structured questionnaire. We examined the pre- and post-course proportions of correct responses to questions, along with the mean score difference, categorized by learners' sex, age, and geographical area. Eventually, a Satisfaction Training Index was created.

**Results:**

The maximum capacity was reached, with 29,998 out of 30,000 available spots filled. Of those enrolled, 18,282 HCPs successfully completed the training. Post-test results revealed an increase in correct answers across all MCQs, with overall mean score rising from 48.8 to 68.0 (*p* < 0.001). Stratified analysis indicated improvements across all participant categories. A higher average increase among female (19.87) compared to male enrollees (17.06) was detected (*p* < 0.001). Both “over 55” and “46–55” age groups showed the greatest score increases compared to “35–46” and “under 35” groups, despite no significant differences in pre-test scores. Course satisfaction was high, averaging 4.38 out of 5. Top-rated aspects included “learning new concepts” (4.49), “accessibility” (4.46), and “platform functionality” (4.46).

**Conclusion:**

Our research hypothesis was confirmed by the significant increase in knowledge going from pre-test to post-test and by the high level of user satisfaction. The obtained results serve as a foundation for planning additional professional education in TGD health.

## 1 Introduction

Transgender and gender diverse (TGD) people represent a broad spectrum of individuals whose gender identities differ from the assigned sex at birth (1). At the beginning of the twentieth century, TGD identities were often viewed through a pathological lens. However, the global medical community has since reclassified TGD identities as not being mental disorders. In the American Psychiatric Association's Diagnostic and Statistical Manual, Fifth Edition (DSM-5), published in 2013, the diagnosis of gender dysphoria pertains to the distress and discomfort associated with being TGD, rather than the gender identity itself (2). Subsequently, the World Health Organization's International Classification of Diseases, Eleventh Edition (ICD-11), released in 2019, includes gender incongruence in a chapter on sexual health, emphasizing the individual's experienced identity and the necessity for gender-affirming treatment arising from that identity (3).

Some TGD individuals—but not all—choose to modify their bodies to align more closely with their gender identity through a gender-affirming pathway that progresses in stages. This process may include hormonal treatment and/or surgical interventions (1, 4). It is crucial to highlight that the healthcare needs of the TGD population extend beyond the medical process of gender affirmation, which has been the primary focus of scientific literature to date. Indeed, there are general healthcare needs (e.g., participation in cancer screenings, etc.) that are similar to those of the cisgender population, although they require specific preparation. In line with what has been stated, international recommendations advocate for an inter- and multidisciplinary approach to TGD healthcare, involving professionals from various fields to support gender-affirming interventions as well as preventive care and chronic disease management (1). Unfortunately, TGD individuals still face significant healthcare challenges, resulting in health disparities compared to the cisgender population (4–8). These disparities, affecting both physical and mental wellbeing, arise from discrimination, systemic biases limiting healthcare access, and lack of knowledge and skills among healthcare professionals (HCPs) in delivering competent care to TGD individuals (4–6, 9). Indeed, across diverse disciplines, educational programs at all stages, spanning undergraduate, graduate, residency, and continuing education levels, have typically disregarded cultural or clinical training pertaining to TGD individuals (1, 10). Various international studies, mainly conducted in the US, Canada, and Europe, have reported instances of TGD individuals feeling estranged in healthcare settings due to a lack of knowledge among HCPs (8, 9, 11–15). Consequently, TGD individuals often avoid seeking healthcare, resulting in negative consequences like poor adherence to cancer screening, delayed disease diagnosis, and self-medication without proper supervision (6, 16, 17).

Underscoring the significance of training HCPs in TGD health is essential, as it is recognized as crucial not only by the TGD community but also by the HCPs themselves. Indeed, while previous studies reveal that only a minority of healthcare personnel have undergone training on TGD health (18–22), many of HCPs regard focused training as one of the most pressing measures necessary to enhance the wellbeing of this population (18, 23).

Although recommendations from international institutions and scientific organizations highlight the importance of HCPs' expertise in TGD issues (1, 24, 25), efforts to incorporate them into both undergraduate and graduate education are still nascent (1, 26). Furthermore, there is still limited evidence on the most effective way to deliver this training (27, 28); what we know is that all too often, this education is provided in a generic manner within courses dedicated to the health of the LGBT+ population, rather than being specifically targeted at addressing the health needs of the TGD population (26). To bridge this gap, and in alignment with the “Italian Plan for the Implementation and Dissemination of Gender Medicine” (29), the Reference Center for Gender Medicine and the Training Office, both located at the Istituto Superiore di Sanità (ISS)—National Institute of Health in Italy, in collaboration with the Ufficio Nazionale Antidiscriminazioni Razziali (UNAR)—National Office against Racial Discrimination, conducted a project aimed at developing a distance learning course targeting all healthcare professions, covering a comprehensive array of topics, such as gender identity, language, TGD health issues, best practices in serving TGD patients, and pertinent legislation. To the best of our knowledge, this was the first such institutional course in Italy and one of the few established at an international level. The objective of this study was to report the results regarding the effectiveness of the course in terms of knowledge improvement and the satisfaction levels of the participants.

## 2 Materials and methods

### 2.1 Elaboration of the scientific contents of the course

With a commitment to developing a course characterized by a multidisciplinary approach that carefully addressed the needs of both HCPs and TGD individuals, a scientific board was established. Comprising recognized experts in TGD healthcare and legal domains, along with representatives from TGD organizations, the primary objective of this board was to identify thematic areas to be covered in the tailored training program for the target audience. Considering that the course represented the first institutional initiative of its kind in Italy, the scientific board deemed it appropriate to formulate a program targeting all healthcare professions, encompassing various aspects related to the care of TGD individuals, and taking into account diverse gender identities. In alignment with the aforementioned goal and considering the available scientific literature on the subject, the following topics were selected: components of sexual identity and its biological foundations, aspects pertinent to psychological support for individuals with gender incongruence in both developmental and adult stages (with a particular focus on communication between HCP and user), best practices for hormonal treatment in individuals with gender incongruence in both developmental and adult stages, fundamental aspects of the gender affirmation surgical pathway, and general principles of the right to gender identity in Italian law.

These themes were translated into the following specific Learning Objectives (LOs).

LO1. Describe the components of sexual identity and its biological bases;

LO2. Identify the aspects useful for providing psychological support to individuals experiencing gender incongruence, both in developmental stages and adulthood;

LO3. Identify best practices for gender affirming hormone therapy, spanning across developmental stages and adulthood;

LO4. Outline the fundamental aspects of gender affirmation surgery;

LO5. Understand the general principles of gender identity rights according to Italian law.

Additionally, a brochure focusing on Sexually Transmitted Infections was developed.

### 2.2 Course characteristic, learning methodology, and participants

The distance course “Transgender Population: From Health to Rights” was accessible free of charge on the ISS e-learning platform (EDUISS, www.eduiss.it). The language utilized in the course was Italian. The course was structured according to Problem-Based Learning (PBL), a methodological approach based on andragogic principles, that stimulates participants to “learn to learn” solving real-world problems that reflect their work context (30). PBL was chosen due to its learning success (e.g., in critical thinking) and its increased usage as a teaching method, especially in medical and healthcare education (31). Over the years PBL has been adapted to the e-learning context and different learning models have been developed, depending on the level of interaction among participants and facilitators (32, 33).

In courses with high turnout, the participants follow the steps of the PBL on their own, without the presence of a facilitator. In this course, the entire PBL cycle (consisting in seven steps) was set up using Totara tools (forum, feedback, sharable content object reference model, file, book, folder, web pages, quiz, and certificate). The first steps of the PBL cycle, consisting of problem analysis and LOs identification, were provided through an interactive tool that allowed to track the results provided by participants.

The course, open to all healthcare professions, was delivered from March 27th, 2023, to September 22nd, 2023. The maximum number of subscribers was 30,000. Successful completion of the course included the release of 16 Continuing Medical Education (CME) credits. The estimated time required to complete all learning activities and the entire course was 16 h. Participants had access to the platform at any time of the day (24 h). According to the Italian regulation, ethics approval was not required for this study: by registering for the course on the online platform, the participants gave the consent to the use of their anonymous data.

The course was structured in four sections:

1. Introductive section: introduction to the course explaining its relevance, general aims and structure, general objectives of the course, guide for participants containing all the instructions to attend the course, Attitudes, Skills and Practices Questionnaire (ASPQ) at time T0, preliminary formative assessment test (pre-test) to set the initial knowledge with Multiple Choice Questions (MCQs). No minimum score was required to complete the pre-test.
2. PBL cycle with the following resources: a problem presentation, a Sharable Content Object Reference Model (SCORM) exercise for analyzing the problem and identifying specific LOs, research and study of materials (bibliographical references and a list of useful websites to be consulted, reading materials to deepen the topics of the course, and audio-video tutorials by experts), and the problem solution.
3. Conclusive section: ASPQ at time T1, formative assessment test (post-test) to set the acquired knowledge with the same MCQs set of the pre-test, final certification test, Satisfaction Questionnaire (SQ). The SQ, which was optionally fillable, could be accessed by users who had completed the learning unit. Passing the final certification test, consisting of 48 MCQs, was mandatory to complete the course and get the CME credits. The final certification test was passed with a score of at least 75% correct answers. Three passing attempts were allowed.
4. Follow-up: it includes ASPQ and formative assessment test at T2 (6 months after the end of the course, and ongoing at the time of writing).

It has been observed that MCQs may not be entirely suitable for evaluating the skills developed through PBL, as assessment ideally should gauge performance rather than simply correct responses (34). However, MCQ tests can be deemed appropriate for formative assessment, particularly when assessing a large volume of knowledge, as in the present case (35). The utilization of MCQ tests was also chosen since the evaluation targeted the levels of “understanding” and “remembering” (36). It was mandatory to answer all questions in each MCQ test.

### 2.3 Data collection

When registering for the course on the e-learning platform, the following demographic and professional information about the participants was collected: sex, age, place of residence, CME profession and discipline, and professional status.

Before accessing the training resources two tools were administered:

- ASPQ. The Questionnaire consisted of batteries of items using Likert scale to detect attitudes, skills and practices;
- Pre-test. The test comprised 10 MCQs (two questions for each LO), aimed at acquiring insights into various areas of knowledge.

At the end of the course the following tools were administered:

- The same set of 10 MCQs was administered (post-test), prior to the CME certification exam;
- The same ASPQ was administered to assess changes over time;
- Participants who completed the learning unit were required to fill in the SQ, consisting of a battery of 18 items using a Likert scale from 1 (minimum level of agreement) to 5 (maximum level of agreement) and two open questions to collect the positive aspects and suggestions for improving the quality of the course.

### 2.4 Statistical analysis

All data were extracted from EDUISS platform. We performed a descriptive analysis (absolute numbers and percentages) to represent demographic, professional, and course completion status information. The pre-test and post-test results were reported as percentages of correct answers to each question and compared through the McNemar test. Data were stratified by sex, age categories and place of residence (Northwest Italy, Northeast Italy, Central Italy, South Italy, and Islands). A new variable denominated Score Increase (SI) was created using the difference between the post- and pre-test individual scores. To identify any differences in scores based on the variables under consideration, one-way analysis of variance (ANOVA) and *post-hoc* tests with Bonferroni method were carried out. We analyzed the satisfaction data taking into account all users who completed the SQ (*n* = 20,450), rather than only those who finished the course (*n* = 18,282), in order to mitigate the possibility of overestimating satisfaction. A Satisfaction Training Index (STI) was created using the average scores of the 18 items of the satisfaction scale. In particular, all satisfaction items were subjected to Pearson correlation that showed a large correlation (>0.6) (37). All items were also subjected to principal axis factorization analysis, which revealed a single factor that reproduces 69% of the total variability, thus the reliability of the unidimensional scale was assessed using Cronbach's alpha. The findings indicate excellent reliability of the instrument, with Cronbach's alpha value exceeding the index of 0.9 (38). To identify any differences in satisfaction based on participants' characteristics, ANOVA and *post-hoc* tests with Bonferroni method were carried out. Statistical analysis was performed using IBM SPSS Statistics 28.0. The results were considered statistically significant at *p* < 0.05.

## 3 Results

### 3.1 Course participants

The training was intended for all CME professions and disciplines and a maximum number of 30,000 members was expected. The maximum capacity has been reached, with a total of 29,998 participants enrolled. All participants were from Italy. The number of participants who completed the course (completers) was 18,282 (60.9%) out of the total enrolled users (Table 1); this proportion raised to 69.4% when excluding users who registered but never entered the course (3,667).

**Table 1:** Characteristics of participants.

	**Completers *n* (%)**	**All registered users *n* (%)**
**Sex**		
Male	4,855 (26.6)	7,842 (26.1)
Female	13,427 (73.4)	22,155 (73.9)
**Age (years)**		
Up to 35 years	3,560 (19.5)	6,004 (20.0)
36–45 years	3,699 (20.2)	6,316 (21.1)
46–55 years	6,300 (34.5)	10,276 (34.3)
Over 55 years	4,723 (25.8)	7,401 (24.7)
**Place of residence**		
Northwest	5,331 (29.2)	8,347 (27.8)
Northeast	1,950 (10.7)	2,989 (10.0)
Central	4,677 (25.6)	7,804 (26.0)
South	4,231 (23.1)	7,368 (24.6)
Islands	2,070 (11.3)	3,447 (11.5)
Abroad	23 (0.1)	42 (0.1)
**Health professions**		
Surgeon	1,526 (8.3)	2,321 (7.7)
Dentist	86 (0.5)	132 (0.4)
Pharmacist	236 (1.3)	415 (1.4)
Veterinary	27 (0.1)	53 (0.2)
Psychologist	1,610 (8.8)	2,414 (8.0)
Biologist	355 (1.9)	532 (1.8)
Chemist	130 (0.7)	170 (0.6)
Physicist	22 (0.1)	27 (0.1)
Rehabilitation health professions	2,405 (13.2)	3,851 (12.9)
Professional educator	658 (3.6)	1,044 (3.5)
Physiotherapist	1,258 (6.9)	1,996 (6.7)
Speech therapist	168 (0.9)	277 (0.9)
Orthoptist/ophthalmology assistant	70 (0.4)	110 (0.4)
Podiatrist	21 (0.1)	34 (0.1)
Psychiatric rehabilitation technician	111 (0.6)	190 (0.6)
Developmental neuro and psychomotor therapist	65 (0.4)	114 (0.4)
Occupational therapist	54 (0.3)	86 (0.3)
Preventive health professions	331 (1.8)	534 (1.8)
Healthcare assistant	191 (1.0)	317 (1.1)
Environmental and workplace prevention technician	140 (0.8)	217 (0.7)
Nursing health professions	9,558 (52.3)	16,408 (54.7)
Nurse	9,153 (50.1)	15,769 (52.6)
Midwife	405 (2.2)	639 (2.1)
Healthcare technical professions—technical assistance area	217 (1.2)	352 (1.2)
Dietician	73 (0.4)	109 (0.4)
Dental hygienist	56 (0.3)	98 (0.3)
Hearing care technician	48 (0.3)	71 (0.2)
Cardiovascular pathophysiology and cardiovascular perfusion technician	27 (0.1)	52 (0.2)
Orthopedic technician	13 (0.1)	22 (0.1)
Healthcare technicians—diagnostic technical area	1,779 (9.7)	2,788 (9.3)
Audiometrist technician	26 (0.1)	43 (0.1)
Neurophysiopathology technician	70 (0.4)	96 (0.3)
Medical radiology technician	781 (4.3)	1,249 (4.2)
Biomedical laboratory health technician	902 (4.9)	1,400 (4.7)
**Professional status**		
Private health facilities/NHS employees	14,487 (79.2)	23,918 (79.7)
Private contractors with NHS	396 (2.2)	620 (2.1)
Freelancer	2,654 (14.5)	4,284 (14.3)
Unemployed	744 (4.1)	1,172 (3.9)
Other	1 (< 0.1)	3 (< 0.1)
**Overall**	**18,282 (60.9)**	**29,997 (100.0)**

The overall data are highlighted in bold.

Among the completers, 4,855 (26.6%) were males, and 13,427 (73.4%) were females. The mean age was 47.1 years, with the most represented age group being between 46 and 55 years (34.5%). Considering the place of residence, 29.2% of completers were from the Northwest of Italy, followed by the Central (25.6%), and the Southern (23.1%) areas. Around 11% of completers came from the Northeast and the Islands. In terms of professional status, most participants were NHS employees or employees of private health facilities (79.2%).

### 3.2 Effectiveness of the course

The results of the pre-test vs. post-test comparison are reported in Tables 2, 3. Table 2 displays the percentage of correct answers to test questions associated with each LO. We observed a significant improvement across all 10 questions (*p* < 0.001), as determined by the McNemar test. Particularly noteworthy are the questions linked to LO1 (i.e., the components of sexual identity and its biological bases), LO3 (i.e., best practices for gender affirming hormone therapy), and LO5 (i.e., general principles of gender identity rights), which exhibited a substantial increase in knowledge from pre- to post-test. Moreover, users initially demonstrated a stronger familiarity with topics related to LO2 (i.e., aspects useful for providing psychological support) and LO4 (i.e., fundamental aspects of gender affirmation surgery). Table 3 displays the results of the comparison between average pre-test and post-test scores according to participants' characteristics using *t*-test for paired data. The average overall pre-test and post-test scores were 48.8 and 67.9, respectively (*p* < 0.001), with a mean SI of 19.1. In stratified analysis, a significant improvement in average scores between pre- and post-test was recorded for all the categories considered. When considering SI, comparison by sex showed a higher average increase among females (19.8) compared to males (17.1), *p* < 0.001. Females also reported a higher average pre-test score than males (49.2 vs. 47.7, *p* < 0.001). ANOVA and *post-hoc* test by place of residence showed a higher score increase among completers in the Northwest (21.2) and Northeast (23.2), *p* < 0.001, compared to other geographical area. *Post-hoc* tests showed that users residing in northern areas obtained pre-test higher scores than users from other geographical areas.

**Table 2:** Knowledge level of completers before and after the course.

		**Correct answerspre-test*n* (%)**	**Correct answerspost-test*n* (%)**	**SI**	***P*-value**
LO1—Describe the components of sexual identity and its biological bases	Q1	7,449 (40.7)	10,251 (56.1)	15.4	< 0.001
	Q2	5,389 (29.5)	7,874 (43.1)	13.6	< 0.001
LO2—Identify the aspects useful for providing psychological support to individuals experiencing gender incongruence, both in developmental stages and adulthood	Q3	10,920 (59.7)	13,003 (71.1)	11.4	< 0.001
	Q4	11,946 (65.3)	12,414 (67.9)	2.6	< 0.001
LO3—Identify best practices for gender affirming hormone therapy, spanning across developmental stages and adulthood	Q5	8,187 (44.8)	11,154 (61.1)	16.3	< 0.001
	Q6	3,451 (18.9)	6,698 (36.6)	17.7	< 0.001
LO4—Outline the fundamental aspects of gender affirmation surgery	Q7	13,517 (73.9)	14,190 (77.6)	3.7	< 0.001
	Q8	15,324 (83.8)	15,424 (84.4)	0.6	< 0.001
LO5—Understand the general principles of gender identity rights according to Italian law	Q9	6,115 (33.4)	12,268 (67.1)	33.7	< 0.001
	Q10	6,991 (38.2)	9,146 (50.0)	11.8	< 0.001

The number of participants who completed the course (completers) was 18,282. Data were compared through the McNemar test. SI was calculated using the difference between the post- and pre-test scores.

LO, Learning Objective; Q, Question; SI, Score Increase.

**Table 3:** Comparison of pre-test and post-test average scores among completers based on participants' characteristics.

	**Pre-test mean score**	**Post-test mean score**	**SI**	***P*-value**
**Sex**				
Male (4,855)	47.73	64.79	17.06	< 0.001
Female (13,427)	49.24	69.12	19.87	< 0.001
**Age (years)**				
Up to 35 years (3,560)	49.95	67.33	17.38	< 0.001
36–45 years (3,699)	49.02	66.96	17.95	< 0.001
46–55 years (6,300)	48.06	67.89	19.82	< 0.001
Over 55 years (4,723)	48.91	69.35	20.44	< 0.001
**Place of residence**				
Northwest (5,331)	50.09	71.30	21.21	< 0.001
Northeast (1,950)	49.61	72.83	23.21	< 0.001
Central (4,677)	48.47	66.12	17.64	< 0.001
South (4,231)	47.26	64.35	17.09	< 0.001
Islands (2,070)	48.95	66.37	17.41	< 0.001
Abroad (23)	50.00	60.00	20.00	< 0.001
**Health professions**				
Surgeon (1,526)	54.22	72.73	18.51	< 0.001
Dentist (86)	49.77	60.12	10.35	< 0.001
Pharmacist (236)	47.29	65.72	18.43	< 0.001
Veterinary (27)	53.70	69.63	15.93	< 0.001
Psychologist (1,610)	54.75	77.29	22.53	< 0.001
Biologist (355)	49.16	68.82	19.66	< 0.001
Chemist (130)	46.54	72.46	25.92	< 0.001
Physicist (22)	46.82	71.36	24.55	< 0.001
Rehabilitation health professions (2,405)	46.85	65.11	18.26	< 0.001
Preventive health professions (331)	46.61	65.39	18.79	< 0.001
Nursing health professions (9,558)	47.79	67.10	19.31	< 0.001
Healthcare technical professions—technical assistance area (217)	51.06	68.34	17.28	< 0.001
Healthcare technicians—diagnostic technical area (1,779)	47.57	64.54	16.97	< 0.001
**Professional status**				
Private health facilities/NHS employees (14,487)	48.23	67.35	19.12	< 0.001
Private contractors with NHS (396)	51.44	71.21	19.77	< 0.001
Freelancer (2,654)	51.55	70.78	19.23	< 0.001
Unemployed (744)	49.73	68.33	18.60	< 0.001
Other (1)	-	-	-	-
**Overall (18,282)**	**48.84**	67.97	19.13	< 0.001

Data were analyzed using paired t-test. SI was calculated using the difference between the post- and pre-test scores. The overall data are highlighted in bold.

SI, Score Increase.

Furthermore, “over 55” and “46–55” completers age groups showed the highest score increase compared to the “35–46” and “under 35” completers group, although there were no significant differences in the pre-test score between the age groups.

### 3.3 Satisfaction Questionnaire results

**Figure 1:**
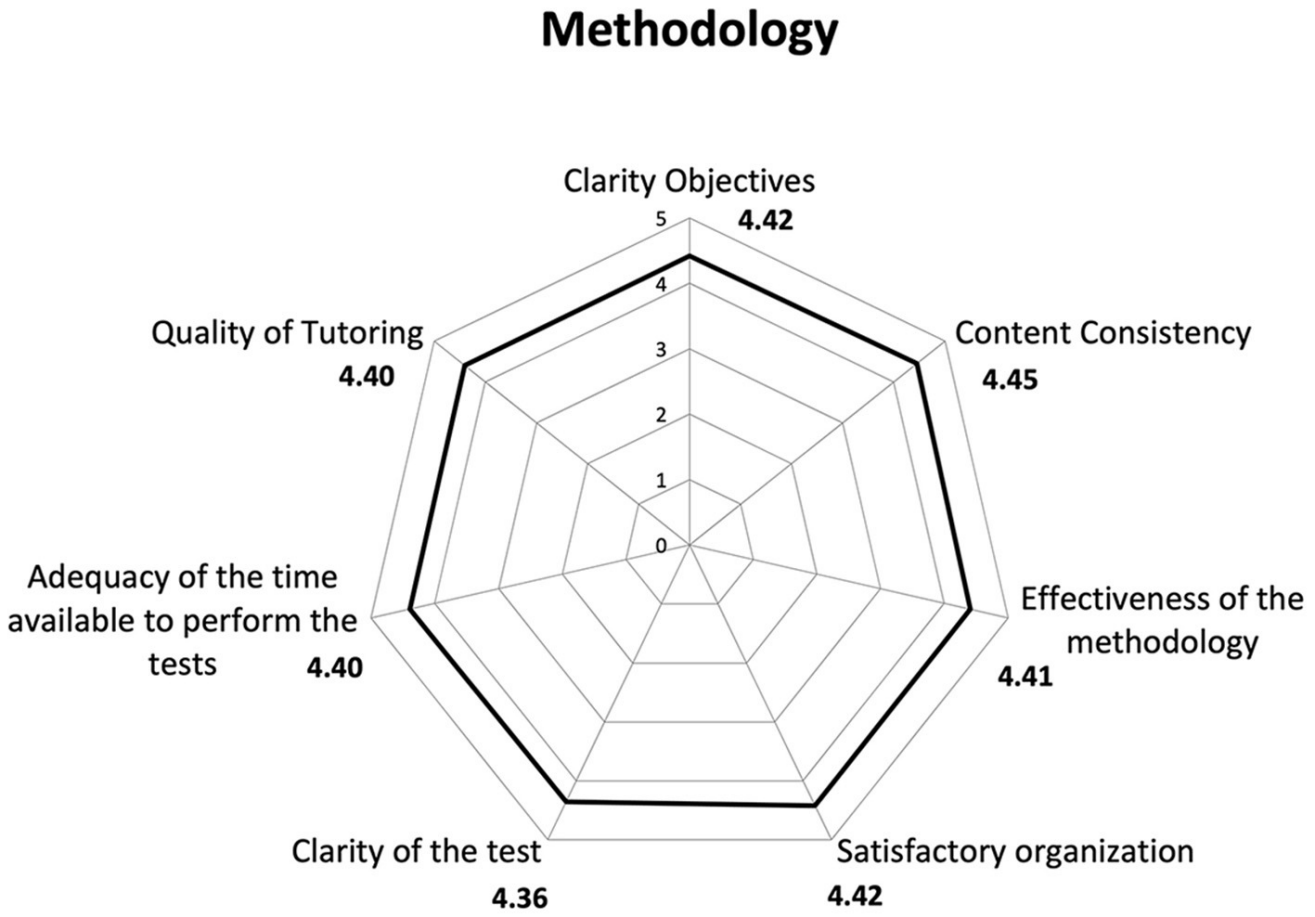
Results of Satisfaction Questionnaire on the learning methodology of the course. We analyzed the satisfaction data taking into account all users who completed the Satisfaction Questionnaire (*n* = 20,450).

**Figure 2:**
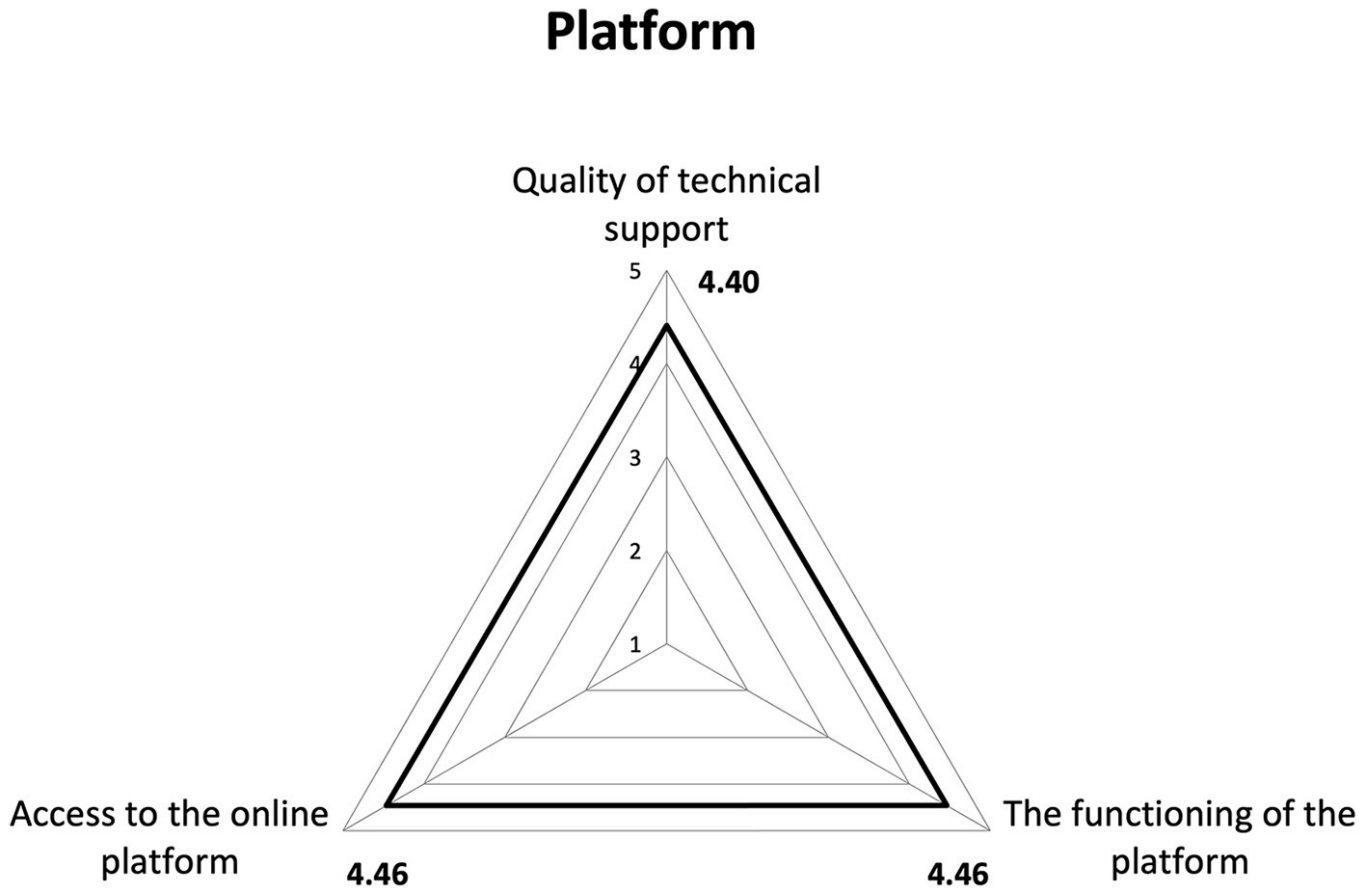
Results of Satisfaction Questionnaire on e-learning platform. We analyzed the satisfaction data taking into account all users who completed the Satisfaction Questionnaire (*n* = 20,450).

**Figure 3:**
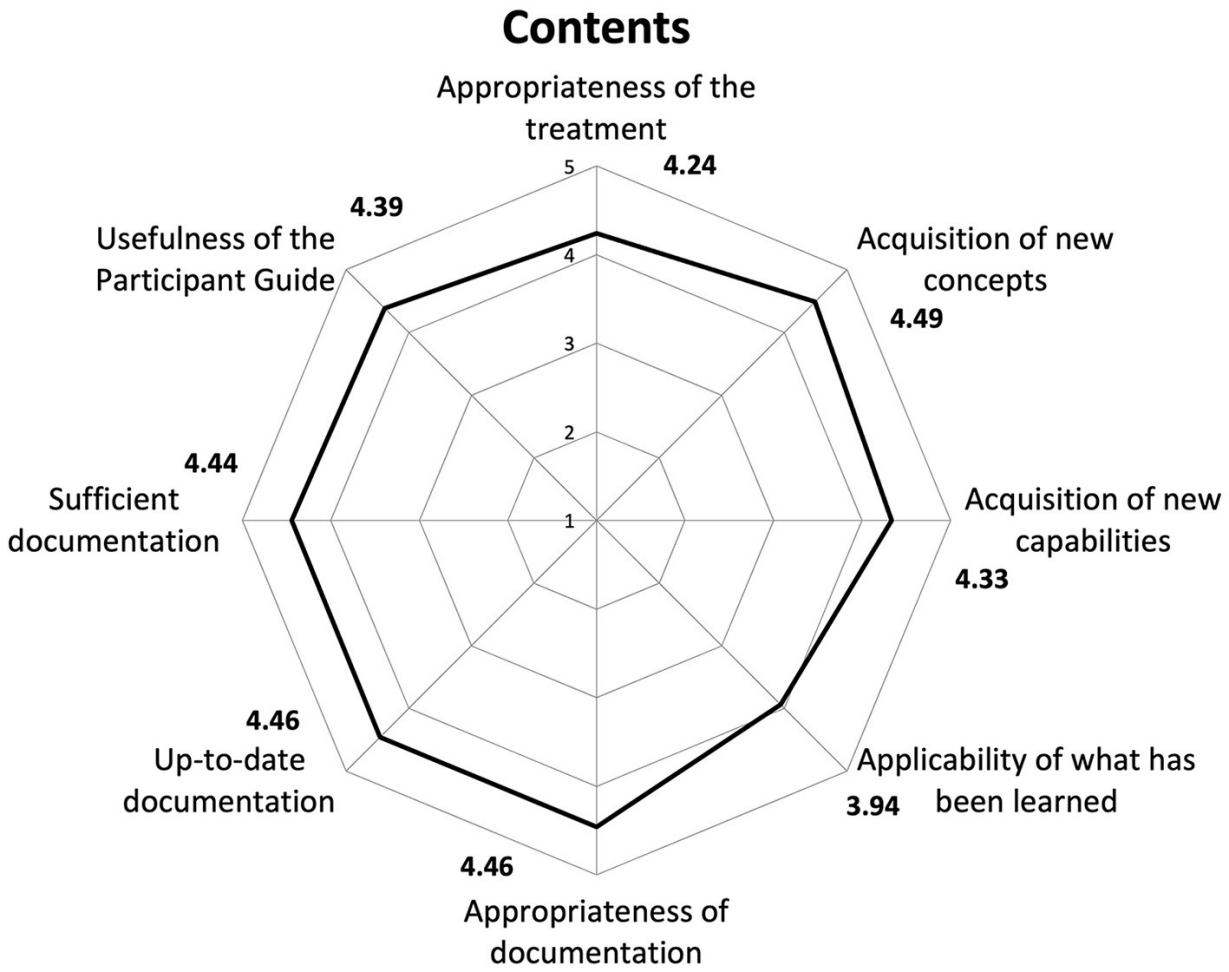
Results of Satisfaction Questionnaire on contents. We analyzed the satisfaction data taking into account all users who completed the Satisfaction Questionnaire (*n* = 20,450).

STI revealed an average satisfaction of 4.38 in all those who filled out the questionnaire. Consequently, a high overall approval of the course emerged, considering that the attributed scores were >4, with 5 representing the highest level of satisfaction (Figures 1–3).

ANOVA by place of residence and Bonferroni *post-hoc* test showed that participants residing in the Islands reported a higher level of satisfaction (4.46), compared with participants from the Northwest (4.37), Central (4.38), and Southern Italy (4.37), *p* < 0.001. Lower satisfaction was observed among younger participants in comparison to other age groups, while no significant differences were found based on sex.

Regarding satisfaction with different aspects of the training, items that received scores closer to five included “learning new concepts” (4.49), “accessibility” (4.46), and the “functionality of the platform” (4.46). In contrast, a slightly lower score (3.94) was recorded for the item on participants' perception regarding the applicability of what they learned in the course to their professional context.

## 4 Discussion

The course “Transgender Population: From Health to Rights” represents the first Italian institutional experience in training HCPs on TGD health. The primary innovative feature of this course lies in its teaching methodology, emphasizing active training. The PBL approach employed encourages participants to engage in “learning to learn” by tackling real-world problems that mirror their professional contexts (30, 39). According to Schmidt et al. (30), PBL leverages the presentation of a problem to stimulate participants' existing knowledge, facilitating more effective learning. In contrast to traditional methods, PBL requires participants to actively address and solve problems, taking charge of identifying their own LOs. Consequently, learners encounter cognitive conflicts and construct their understanding based on prior knowledge and experiences (40). Additional strengths of the course included its large participant base and its comprehensive approach to TGD health, which not only covered every aspect of the subject but also emphasized the importance of appropriate communication between healthcare professionals and the community, while simultaneously addressing the social and legal barriers that contribute to health inequalities.

The results of our course offer a foundation for understanding the effectiveness and reach of the training initiative. Overall, the high participation indicates a significant interest in the training across CME professions and disciplines. The significant concentration of participants aged 46–55 raises interesting questions about the training's appeal to mid-career professionals. Typically, individuals within the older demographic are noted to display heightened enthusiasm for online learning, attributed to their perceived higher self-efficacy and enhanced mental preparedness (41). Moreover, within our study, it is plausible that professionals in the 46–55 age bracket might have encountered fewer opportunities for exposure to or formal education on subjects like TGD health during their earlier training years. Conversely, in today's digital age, younger professionals often encounter diverse perspectives and emerging issues like TGD health more readily through their interactions on social media platforms and engagement with online communities. Understanding what attracts participants from different age groups can help tailor the course content, format, and outreach efforts to better meet the needs and interests of a diverse range of HCPs.

The outcomes derived from our course demonstrate a notable enhancement in the knowledge levels of participants, as evidenced by the comparison between pre-test and post-test results across all categories. This suggests that individuals with diverse backgrounds and characteristics derived substantial benefits from the course, affirming its effectiveness in reaching a broad audience. This emphasizes the importance of establishing a foundational knowledge on TGD health that is shared across various health professions. Sex-based analysis revealed a higher average increase among female participants compared to their male counterparts. The observation that females also reported a higher average pre-test score suggests a potential baseline difference in attitudes to transgender health topics (42, 43), further emphasizing the importance of tailoring educational interventions to address diverse starting points among participants.

In addition, the age-based analysis revealed that participants aged over 55 and those aged 46–55 demonstrated the highest score increase compared to younger age groups, despite no significant differences in the initial scores between age groups being detected. This finding is consistent with previous research indicating that older students tend to achieve higher grades than their younger counterparts (41, 44). Moreover, our study suggests that mid-career and older participants, who were more numerous as previously mentioned, may have been particularly motivated and engaged in the course. This heightened motivation and engagement could have contributed to the observed score improvements.

Analysis of the distribution of correct responses to pre-test and post-test questions highlighted some points of interest. Concerning aspects useful for providing psychological support (LO2) and the fundamental aspects of gender affirmation surgery (LO4), users showed a greater propensity to provide a correct answer during the pre-test. This result may reflect existing awareness or knowledge among participants regarding these important aspects of transgender health, although it's also plausible that the questions related to these topics were relatively less complex.

Conversely, concerning the description of components of sexual identity and its biological underpinnings (LO1), best practices for gender-affirming hormone therapy (LO3), and the general principles of gender identity rights (LO5), the initial assessment indicated a more pronounced educational gap. However, encouragingly, participants exhibited substantial improvement in their understanding of these topics by the conclusion of the course, as evidenced by the post-test results. These findings underscore the effectiveness of the course curriculum in addressing educational gaps and fostering knowledge acquisition among participants. Moreover, they provide valuable insights for enhancing question design in future iterations of the course. By identifying areas of strength and weakness in participant understanding, educators can refine course content and assessments to optimize learning outcomes and better meet the educational needs of the target audience.

In terms of participant satisfaction, our findings revealed a positive overall response. This high level of approval is noteworthy, signifying a strong endorsement of the course among the participants. It is worth noting that younger participants expressed lower satisfaction levels, suggesting a potential misalignment between course content and the expectations or requirements of this demographic. This highlights the importance of considering not only the knowledge level but also the satisfaction and engagement of participants when tailoring effective training programs. Collecting feedback from younger participants through surveys, focus groups, or individual interviews can provide invaluable insights into their specific needs, preferences, and areas for improvement.

Analyzing satisfaction with different aspects of the training, several key aspects stood out. Learning new concepts, access to the course, and the functioning of the platform received high scores, indicating that participants found these elements particularly beneficial and well-executed. Conversely, a slightly lower score was recorded for the item assessing participants' perception of their ability to apply what they learned in the course to their working context. This finding suggests an area for potential improvement, with implications for the design and implementation of future TGD health courses. Incorporating clinical competencies related to TGD individuals across the entire curriculum of HCPs, rather than in one or a few condensed lectures, is a priority to be addressed at the institutional level (1).

Our course represents the first institutional attempt to provide HCPs with the basic knowledge related to the health needs of the TGD population. Therefore, the course was designed to be accessible to all healthcare professions, as classified by the Agenzia Nazionale per i Servizi Sanitari Regionali (AGENAS)—National Agency for Regional Health Services. Extending access to all HCPs, including those not directly involved in healthcare assistance, appeared to be a strategic approach to foster a culture of inclusivity for TGD individuals on a wider scale. However, behind this aim lies one of the study's limitations, as the identified constraints reflect weaknesses in the course design. The distribution of enrolled participants mirrors the composition of HCPs accredited in EDUISS, where nurses constitute approximately one-third of the total accredited professionals (with 223,000 nurses out of 616,000 healthcare professionals). Due to this limitation, to ensure robust results when comparing pre-test and post-test scores, we opted to aggregate certain categories within the “profession” variable according to the classification proposed by AGENAS. Consequently, we abstained from providing average pre-test and post-test scores for individual professions. Another limitation of the study is the lack of consideration of follow-up data. As of the time of writing, the course's follow-up is in progress, involving the administration of the formative assessment test (T2) and the ASPQ 6 months after the course's conclusion. These data may be explored in a future study focused on tracking the progression of attitudes, skills, and practices.

## 5 Conclusions

In conclusion, the goal of this study, as well as the overall success of the training program, was evidenced by the high enrollment numbers, which hit the maximum capacity. The research hypothesis was validated by the notable improvement in knowledge from pre-test to post-test and the high degree of user satisfaction. Our results mark a crucial first step in refining and optimizing the delivery of educational programs in TGD health. They underscore the necessity of considering specific demographic factors in the formulation of forthcoming educational strategies in TGD health. Future literature addressing the assessment of learners in clinical settings and the impact on patient outcomes is urgently warranted. The implications of these findings extend beyond the immediate educational context, contributing to the broader discourse on promoting inclusivity and understanding in healthcare practices related to TGD health.

## Data availability statement

The raw data supporting the conclusions of this article will be made available by the authors, without undue reservation.

## Ethics statement

Ethical approval was not required for the studies involving humans because, according to the Italian regulation, ethics approval was not required for this study: by registering for the course on the online platform, the participants gave the consent to the use of their anonymous data. The study was conducted in accordance with the local legislation and institutional requirements. The participants provided their written informed consent to participate in this study.

## Author contributions

UF: Validation, Software, Methodology, Investigation, Formal analysis, Data curation, Conceptualization, Writing – review & editing, Writing – original draft. MMar: Project administration, Methodology, Investigation, Formal analysis, Data curation, Conceptualization, Writing – review & editing, Writing – original draft. PC: Software, Methodology, Formal analysis, Data curation, Writing – review & editing. DG: Software, Methodology, Formal analysis, Data curation, Writing – review & editing. AR: Methodology, Investigation, Writing – review & editing. MMan: Formal analysis, Data curation, Writing – review & editing. FM: Software, Methodology, Data curation, Writing – review & editing. FR: Software, Project administration, Methodology, Data curation, Writing – review & editing. AV: Software, Methodology, Data curation, Writing – review & editing. AM: Supervision, Methodology, Writing – review & editing. MP: Supervision, Resources, Project administration, Investigation, Funding acquisition, Formal analysis, Data curation, Conceptualization, Writing – review & editing, Writing – original draft.
